# Treatment strategies for thromboembolism-in-transit with pulmonary embolism

**DOI:** 10.1093/icvts/ivac183

**Published:** 2022-06-27

**Authors:** Hiroki Sakai, Takayuki Uchida, Takashi Matsumoto

**Affiliations:** Department of Cardiovascular Surgery, Iizuka Hospital, Yoshiomachi, Iizuka, Fukuoka, Japan; Department of Cardiovascular Surgery, Iizuka Hospital, Yoshiomachi, Iizuka, Fukuoka, Japan; Department of Cardiovascular Surgery, Iizuka Hospital, Yoshiomachi, Iizuka, Fukuoka, Japan

**Keywords:** Pulmonary embolism, Endarterectomy, Thrombosis, Cardiac catheterization, Intervention

## Abstract

A 46-year-old obese woman undergoing treatment for bipolar disorder presented with acute shortness of breath, chest pain and palpitations. She was tachypnoea and tachycardia, but blood pressure was stable. Computed tomography angiogram revealed bilateral pulmonary embolism. Echocardiogram revealed thrombus-in-transit. She underwent surgical embolectomy only for thrombus-in-transit and closure of the patent foramen ovale. However, pulmonary hypertension worsened, haemodynamical instability prolonged and hepatic congestion progressed. After veno-arterial extracorporeal membrane oxygenation insertion, we performed thrombectomy by catheter and anticoagulation therapy. One month later, the patient was transferred to another hospital for rehabilitation.

## INTRODUCTION

Thromboembolism-in-transit across the patent foramen ovale (TIT-PFO; impending paradoxical embolism) is a rare condition most often seen in conjunction with deep venous thrombosis and pulmonary embolism (PE). Due to the abrupt rise in pulmonary arterial pressure, the direction of the shunt through the PFO may change right to left and allow migration of the thrombus across the arterial septum to the systemic circulation. This predisposes to potentially fatal complications, embolization to the brain being the biggest threat. Treatment options are antithrombotic therapy, thrombolysis and surgery. The best option has not yet been decided and is prone to individual assessment.

## CASE REPORT

A 46-year-old female was referred to the hospital after 5 days of progressive shortness of breath and chest pain. Her medical history further included obesity and bipolar disorder. She was taking olanzapine, lithium carbonate and bromazepam for bipolar disorder and tended to lie in bed until just before onset. One week before admission, she felt dyspnoea, but it resolved spontaneously. However, it returned, and she came to the emergency room. On admission, she reported dyspnoea class III–IV according to the New York Heart Association (NYHA) classification.

**Figure 1:**
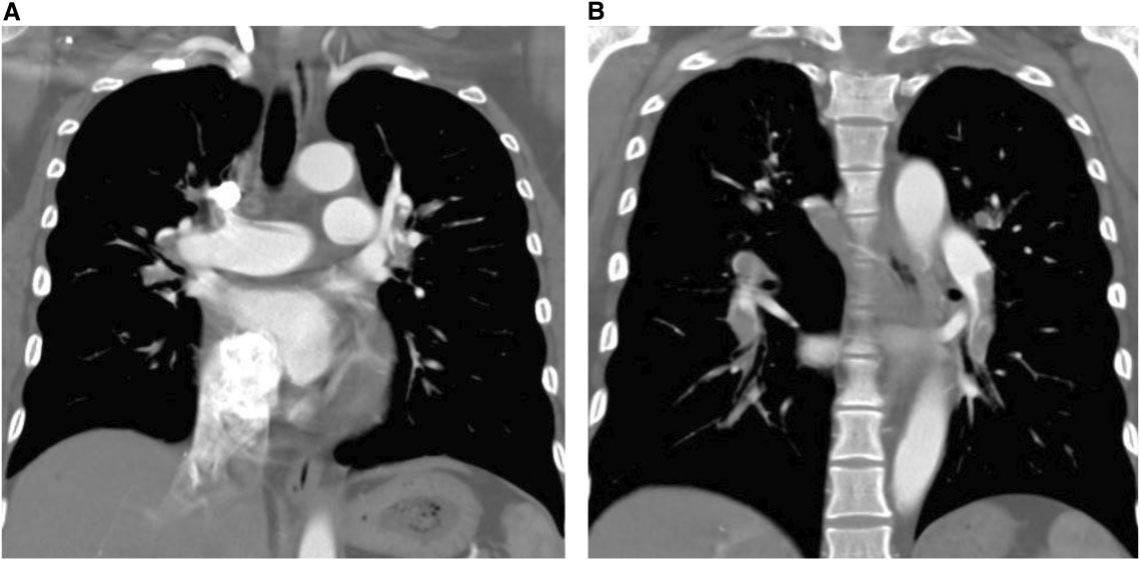
(**A**) Few large thrombi were found in the central pulmonary artery. (**B**) A large thrombus was detected in the peripheral pulmonary arteries.

Physical examination revealed an overweight female with malaise. Height: 153 cm, weight: 83 kg, BMI 35.5 kg/m^2^. Upon arrival, she had tachycardia of 129 beats/min and blood pressure of 103/77 mmHg, pronounced respiratory distress (31/min) and oxygen saturation of 99% with oxygen supply. Clinical examination was otherwise normal. Electrocardiogram confirmed sinus tachycardia, negative T wave in lead III, aVF and V1. Initial arterial blood gas analysis showed pH 7.534, partial pressure of carbon dioxide of 22.8 mmHg, partial pressure of oxygen of 90.3 mmHg, and bicarbonate of 18.8 mmol/l with 2.0 l/min oxygen supply, lactate 26.1 mg/dl. She had a BNP of 690.2 pg/ml and CRP of 2.03 mg/dl. D-dimer was 3.1 μg/ml. Computed tomography scan revealed central bilateral PE branching out to the segmental arteries of the lobes. Few large thrombi were found in the central pulmonary artery (Fig. 1A), but a large thrombus was detected in the peripheral pulmonary arteries (Fig. 1B).

**Figure 2:**
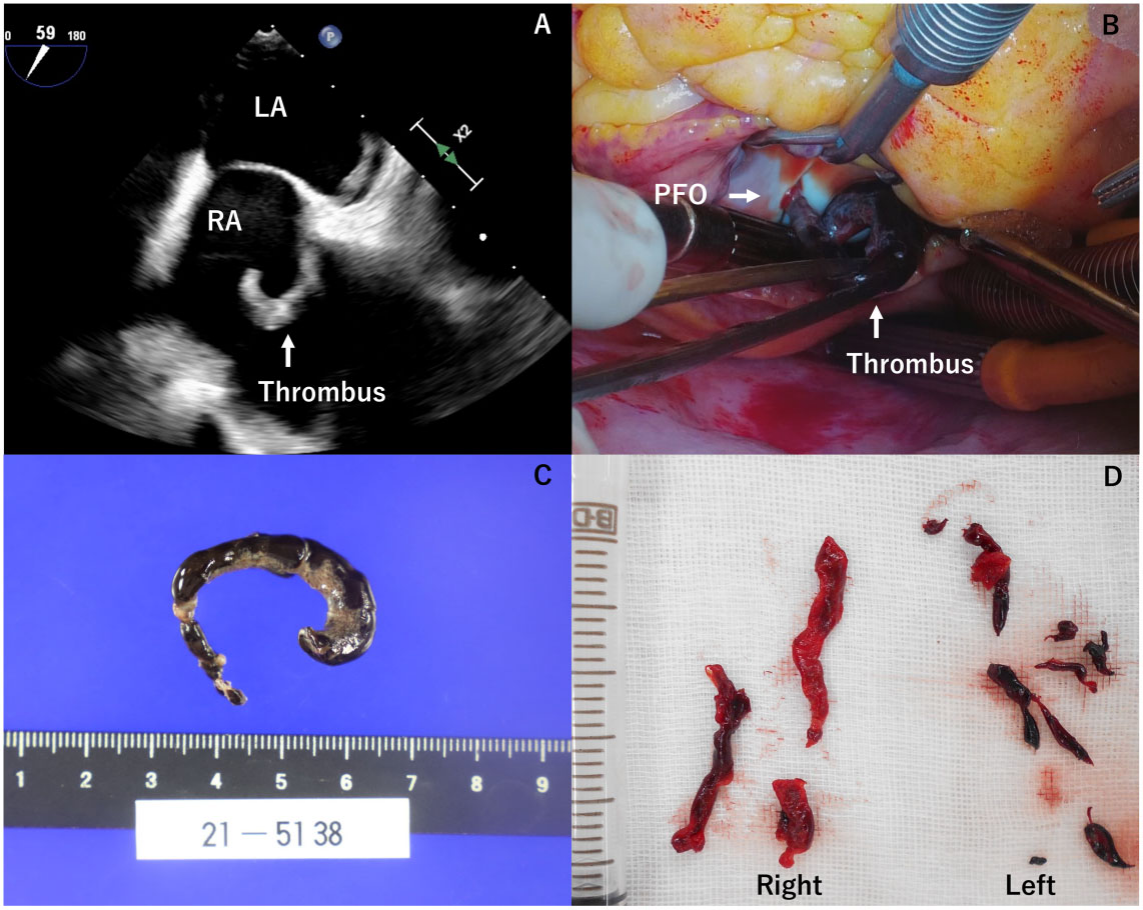
(**A**) Transoesophageal echocardiography, worm-like thrombus (arrows) straddling the PFO in both atria. (**B**) Clot retrieved at embolectomy. A worm-like thrombus was lodged into the PFO. (**C**) Pathologically, the thrombus has no malignant findings. LA: left atrium; PFO: patent foramen ovale; RA: right atrium. (**D**) Clot retrieved at thrombectomy by aspiration using a catheter. White and red thrombi were removed from the right PA. Many red thrombi were removed from the left PA. (A color version of this figure appears in the online version of this article.)

No thrombosis was seen in the superior and inferior vena cava, but some were observed in the iliac vessels. Transthoracic echocardiography was performed showing a slightly dilated right ventricle, but with preserved systolic function. Transoesophageal echocardiogram showed a dysfunctional and dilated right ventricle and a large serpentine, mobile mass in the right atrium, extending to the left atrium through the PFO (Fig. 2A).

Respiratory and circulatory dynamics were stable. We performed thrombectomy in atrium and closure of the PFO to prevent thrombosis in systemic circulation. Under cardiopulmonary bypass, we removed a fresh thrombus straddling across the PFO (Fig. 2B and C) and closed the PFO.

She was haemodynamically stable and transferred to the intensive care unit with an infusion of dobutamine hydrochloride and milrinone. On the next day of surgery, blood tests showed elevated hepatic enzyme, progressed hepatic congestion on abdominal echography and elevated central venous pressure. Veno-arterial extracorporeal membrane oxygenation insertion was performed to improve hepatic congestion. A 21 Fr multistage cannula was inserted in the right femoral vein with its tip in the right atrium. A short 18 Fr catheter was placed in the femoral artery with the tip in the common iliac artery. Anticoagulation therapy was continued for the next 2 days, but pulmonary artery pressure did not decrease. In this condition, weaning of veno-arterial extracorporeal membrane oxygenation was difficult, so pulmonary artery thrombectomy by catheter was performed. Pulmonary arteriography showed acute on chronic pulmonary thromboembolism, so we performed thrombectomy by catheter as far as possible. A huge amount of thrombus was aspirated during the catheter intervention (Fig. 2D). After catheter intervention, pulmonary artery pressure decreased and left ventricular output increased gradually. The patient was able to be weaned off extracorporeal membrane oxygenation because of the decrease in pulmonary artery pressure and improvement in oxygenation within a few days after catheter-assisted thrombus removal (CATR). Five days after CATR, the patient was extubated. One month later, she was transferred to another hospital for rehabilitation.

## DISCUSSION

The annual incidence of deep venous thrombosis including PE is ∼1 per 1000. Based upon autopsies, a PFO is present in 20–30% of the population. Despite this seemingly high likelihood of catching a thromboembolism-in-transit across the PFO, it is seldom encountered in clinical practice. Hui *et al.* [1] suggest that echocardiography should be considered for routine surveillance in thromboembolism because of the risk of systemic sequelae. The abrupt rise in pulmonary arterial pressure may contribute to the migration of the thrombus across the atrial septum to the systemic circulation. If any abnormal structures are seen in the left atrium by TTE in a patient with PE, a transoesophageal echocardiography should be performed to rule out an embolus entrapped in a PFO [2]. Because of its high risk, Lee *et al.* [3] suggest that integral diagnostic workup should be performed in cases of acute pulmonary thromboembolism whether the patient is haemodynamically stable or not.

The treatment of choice for TIT-PFO is controversial. Surgical thromboembolectomy for TIT-PFO has shown a trend towards improved survival and significantly reduced systemic emboli compared with anticoagulation. On the other hand, mortality after pulmonary embolectomy has been reported as 27–41% [4]. In a recent real-world study, mortality occurred in 19.8% of patients undergoing surgical embolectomy for acute PE. This represents a significant improvement compared with traditional outcomes and supports the role of surgery in the multidisciplinary treatment of this high-risk condition [5]. On the other hand, catheter thrombectomy has been used in a small cohort study, suggesting that the clinical outcome of this procedure is comparable to that of surgical thrombectomy [6]. A recent meta-analysis reported a clinical success rate of 86.6% and a serious complication rate of 2.4% [7]. Thus, it goes without saying that surgical thrombectomy is the preferred treatment for severe PEs where maintaining circulatory control is difficult, but catheter-based thrombectomy may also be a safe and effective treatment option. In the present case, we would have preferred to perform surgical pulmonary thrombectomy if possible. Because of the relatively peripheral distribution of the thrombus within the pulmonary artery, it was anticipated that surgical pulmonary thrombectomy would be insufficiently effective. Therefore, the first step was only to perform thrombectomy in atrium to avoid the surgical risk of pulmonary artery thrombectomy, to perform closure of the PFO to prevent thrombosis in the systemic circulation and to add CATR if haemodynamic status was instable or if poor oxygenation or hepatic congestion occurred.

We performed 2 types of CATR. The first was aspiration thrombectomy, in which a catheter is wedged directly into the thrombus and the catheter is removed from the thrombus keeping negative pressure with a syringe in hand. There is a comprehensive report on the method of aspiration thrombectomy, and it is an effective technique [8]. The second was thrombus fragmentation. A pigtail catheter is rotated around the thrombus to break up the thrombus and disperse it to the periphery [9]. The thrombus is not retrieved, but the surface area of the crushed thrombus is increased, which may also strengthen the thrombolytic effect.

Catheter-directed thrombolysis has been shown to improve right ventricular function in patients with PE. In recent years, this has become safer to perform and its effectiveness is being confirmed [10]. However, it increases bleeding risk and many patients with PE have relative and absolute contraindications to thrombolysis. The acute mortality in PE is driven by haemodynamic compromise due to right ventricular failure. Right ventricular enlargement on computed tomography is a strong predictor of mortality in PE [11]. According to a report, this case was high risk because right ventricle/left ventricle was 1.6. To overcome the haemodynamic compromise by PE, catheter thrombectomy was performed and heparin was injected directly from the tip of a Swan–Ganz catheter into the blood clots remaining in the pulmonary artery.

Initial treatment of massive PE is still thrombolysis. However, how to treat a patient with TIT-PFO with a sub-massive PE is up for discussion. One review article concludes with a trend towards improved survival and a significantly reduced risk of systemic embolism and mortality if the patient undergoes operation rather than conservative treatment [12]. The choice of treatment in each patient must be made after thorough discussion in a multidisciplinary heart team.

**Conflict of interest:** none declared.

## Reviewer information

Interactive CardioVascular and Thoracic Surgery thanks the anonymous reviewer(s) for their contribution to the peer review process of this article.

## Notes

The patient has provided permission to publish these features of her case, and the identity of the patient has been protected (27 July 2021).
